# Systems-informed approaches to evaluating population health interventions: Reflections from three case studies

**DOI:** 10.1177/13563890261444326

**Published:** 2026-05-19

**Authors:** Miriam Alvarado, Elizabeth McGill, Natasha Sobers, Stephanie Whiteman, Jean Adams, Madhuvanti M. Murphy

**Affiliations:** 1 IMS Epidemiology, University of Cambridge School of Clinical Medicine, UK; 2 The George Alleyne Chronic Disease Research Centre, University of the West Indies, Bridgetown, Barbados; 3 Department of Public Health, Environments and Society, Faculty of Public Health and Policy, London School of Hygiene & Tropical Medicine, UK; 4 Department of Pre-Clinical and Health Sciences, Faculty of Medical Sciences, University of the West Indies, Bridgetown, Barbados

**Keywords:** evaluation methodology, evaluation studies, population health, systems analysis, systems thinking

## Abstract

There have been many calls for systems-informed approaches to evaluation. Population health interventions, which often focus on improving the upstream determinants of health through large-scale change, seem well-suited to systems-informed approaches to evaluation. The purported benefits and appropriateness of taking a systems-based approach to population health intervention evaluation have been extensively discussed, and there is a growing, but still limited, number of applied case studies that operationalised these calls. Here, we reflect on insights gained from recent experiences across three evaluation case studies: a late-night levy to support local policing (UK), the tiered soft drinks industry levy (UK) and a value-based tax on sugar-sweetened beverages (Barbados). Building on theoretical work in this area, we illustrate through applied examples how a systems-informed approach can help to cast a wider net for potential impacts, be integrated with an effectiveness perspective to produce deeper insights and support greater engagement with non-linearity.

## Introduction

There have been many calls for systems-informed approaches to evaluation, both within the population health field and beyond (Barbrook-Johnson et al., 2021; Gates et al., 2021; Rutter et al., 2017; Skivington et al., 2021). Systems thinking emphasises the importance of dynamic change, non-linearity, feedback loops, adaptation and emergence (Meadows and Wright, 2009). Systems-informed approaches to evaluation may entail using systems tools and methods, such as causal loop diagramming, system dynamics modelling and agent-based modelling, and conceptualising interventions as events within systems (Gates et al., 2021; Hawe et al., 2009; McGill et al., 2021).

Persistently high global rates of non-communicable diseases underscore the need for population health interventions (PHIs), which focus on improving the upstream determinants of health through large-scale change (alongside other types of interventions (Sniehotta et al., 2017)). Since PHIs operate on a scale that makes it challenging to assess using randomised control trials (RCTs) (Craig et al., 2025; Rutter et al., 2017), evaluation teams (including ourselves) tend to use natural experimental evaluations to quantify the impact of such PHIs and assess the effectiveness-oriented question: ‘To what extent does the intervention produce the intended outcomes in real world settings?’ (Skivington et al., 2021). However, answering this question rigorously and well does not necessarily shed light onto why and how an intervention may have operated, nor how and in what ways the system and intervention may have adapted to one another.

This is a major potential shortcoming of an effectiveness-only evaluation perspective for PHIs, particularly since we *expect* system adaptations to occur following the implementation of (by definition) large-scale policy and infrastructure PHIs. Understanding the range and nature of such adaptations would help to improve and strengthen interventions over time (Hawe and Potvin, 2009; Ogilvie et al., 2019).

Over the past decade, we have led and engaged in systems-informed evaluations of three PHIs (Box 1). Here, we reflect on insights gained from recent experiences across three case studies: a late-night levy (LNL) to support local policing (UK), a tiered soft drinks industry levy (UK) and a value-based tax on sugar-sweetened beverages (Barbados). We aim to illustrate through applied examples how a systems-informed approach can enrich population health evaluations. We hope that these reflections are of value particularly to those at the beginning of their own systems thinking journey as they move from the theoretical literature to conducting systems-informed evaluations.

**Box 1.** Three examples of population health interventions that the authors have contributed to evaluating.

**London’s late-night levy (LNL).** Since 2011, local authorities in England and Wales can charge businesses that sell alcohol between midnight and 0600 a levy to support additional policing and enforcement. Each local authority can choose whether to apply the levy at all, what night-time hours it will apply to and what exemptions and reductions they will allow. The levy varies in cost from £299 to £4440 per annum, and at least 70% must be spent on policing. We evaluated the levy in one local authority in London. We first analysed documents and identified four possible hypotheses of how the levy may create change within the night-time economy system. Next, we interviewed stakeholders, observed community safety patrols and analysed annual levy reports to explore the extent to which the four hypotheses played out in practice (McGill et al., 2022). **The UK’s soft drinks industry levy (SDIL)**. In 2016, the UK announced a tiered levy on the manufacturers of sugar-sweetened beverages (SSBs). From 2018, drinks with more than 5 g of sugar per 100 ml were charged 18p per litre, and those with more than 8 g were charged 24p per litre. Alcoholic drinks, milk-based drinks and pure fruit juices are exempt. We created a conceptual systems map describing the potential impacts of the SDIL and used this to inform the design of a multi-component evaluation. We used routine and newly collected data to conduct quantitative, qualitative, modelling and evidence synthesis evaluative components (White, 2017). **Barbados’ SSB tax.** In 2015, the Government of Barbados implemented a 10% value-based tax on SSBs. Prior to engaging with systems thinking, we conducted a quantitative impact evaluation of the SSB tax and demonstrated a decrease in SSB sales (Alvarado et al., 2019). After reviewing these findings, a stakeholder commented: ‘This is great. But what I want to know is, what should we advise the Minister of Health to do now?’. An effectiveness-only evaluation approach did not enable us to comment on how future actions might produce change. However, we had a unique opportunity to revisit this policy. In 2022, the tax rate was doubled, and we designed a second evaluation, grounded in a community-based system dynamics (CBSD) approach (Hovmand, 2014). The evaluation entailed, among other things, convening a group of diverse stakeholders, conducting a group model-building workshop to develop a shared mental model and creating a system dynamics model and interactive interface, guided by stakeholder priorities and ongoing input.

## A systems-informed approach can help to cast a wider net for potential impacts

In all three evaluations (Box 1), we developed theories of change embedded in systems maps and used these to guide analyses beyond the primary intended pathway of change (a ‘systems framing’ approach, as described in McGill et al. (2021). This helped us to identify a wide range of potential impacts, as well as pathways to these impacts.

For example, we developed a conceptual systems map of the SDIL’s potential impacts through consultations with key stakeholders and a Delphi process (White, 2017). Within this map, we included elements we had expected to evaluate, such as price, reformulation, purchasing, consumption and health. However, the map also helped us broaden our scope to include assessing changes in how SSB companies presented themselves in the media, soft drink business turnover and public acceptability.

If systems-informed approaches help us to cast a wider net, the width of the net depends largely on the diversity of perspectives represented. For example, the LNL was designed to reduce the social impacts of acute alcohol intoxication such as injuries, noise and other antisocial behaviour. However, we also considered the views of local business owners who identified potential indirect harms from the levy on the local Business Improvement District. As a result, we broadened our theory of change. This potential impact would not have been considered relevant if we had remained focused solely on the outcomes the intervention designers sought to influence.

While a systems thinking approach encourages evaluators to forefront the identification and analysis of multiple impacts, other approaches and methods (e.g. comprehensive theories of change, not limited to single pathways) may achieve similar aims.

## Systems-informed and effectiveness perspectives can be integrated to strengthen an evaluation

In the second evaluation of the Barbados SSB tax, we moved back and forth between a systems and effectiveness perspective (Skivington et al., 2021) and found that this was both feasible and enriching. For example, we were able to use a causal loop diagram developed in the early stages of the evaluation to surface possible explanations for a puzzling finding in our quantitative impact evaluation. Later, we used findings from the impact evaluation to calibrate a system dynamics model (SDM), which prompted us to add new model structure and hypothesise about an additional system adaptation.

Going beyond the traditional (in population health) effectiveness evaluation by situating the perceived primary outcome within a broader context of multiple intended and unintended impacts, systems adaptations and feedback loops produce deeper insights. At the same time, some evaluation users (e.g. technical officers in ministries of health and finance) told us that to be most useful, a systems-informed evaluation should be delivered alongside an impact evaluation, as this is the dominant type of evidence policymakers expect when assessing PHIs.

Our experience echoes arguments made by others regarding the relationship between taking a systems perspective and an effectiveness one (Hawe et al., 2004), as well as Moore et al.’s call to use systems thinking to both shape evaluation questions and interpret evaluation process and outcome data (Moore et al., 2019).

## A systems-informed approach supports embracing non-linearity

Systems-informed approaches to evaluation uniquely, we think, emphasise the importance of feedback loops and non-linearity. This can be represented either qualitatively (e.g. causal loop diagramming) or quantitatively (e.g. system dynamics modelling).

We identified several reinforcing feedback loops in our evaluation of the LNL. One example was the development of cooperative relationships between police, community safety patrol officers and licenced venue operators. Key to this cooperative relationship was the sharing of information about late-night venues and patrons, which was used to inform future targeting of levy resources. This reciprocal information sharing built trust between systems actors, which in turn increased the ability of levy resources to be used effectively.

System dynamics modelling can be used to operationalise and visualise potential feedback loops further. For example, in the Barbados SSB tax evaluation, we developed an SDM to represent the links between SSB taxation, SSB consumption, diabetes rates, tax revenue and industry responses. The system structure that most closely aligned with observed trends in SSB sales included an industry learning feedback loop, where industry responses grew stronger and more effective over time. This insight had not been previously articulated but resonated strongly with stakeholders and led to rich discussions about ‘what next?’ from both civil society and government perspectives.

## Conclusion

We suggest that systems-informed evaluation of PHIs can help to cast a wider net for potential impacts, be integrated with effectiveness approaches and facilitate greater engagement with non-linearity. While our experience has been focused on PHIs, some of these reflections may also be relevant to evaluations of other types of interventions, such as health interventions operating at the individual level (Sniehotta et al., 2017), or policy and infrastructure interventions focused on climate mitigation and adaptation (Benton et al., 2025). We look forward to seeing how the broader conversation in this area continues to evolve.

## Data Availability

Data sharing is not applicable to this article as no datasets were generated or analysed during the current study.

## References

[bibr1-13563890261444326] Alvarado M Unwin N Sharp SJ , et al. (2019) Assessing the impact of the Barbados sugar-sweetened beverage tax on beverage sales: an observational study. The International Journal of Behavioral Nutrition and Physical Activity 16(1): 13. 10.1186/s12966-019-0776-7 PMC6354371 30700311

[bibr2-13563890261444326] Barbrook-Johnson P Castellani B Hills D , et al. (2021) Policy evaluation for a complex world: Practical methods and reflections from the UK Centre for the evaluation of complexity across the nexus. Evaluation 27(1): 4–17.

[bibr3-13563890261444326] Benton L Brousselle A McDavid J , et al. (2025) Need for planetary health perspective in guidance for complex interventions for climate and health. BMJ 389: e083337. 10.1136/bmj-2024-083337 PMC12326577 40461156

[bibr4-13563890261444326] Craig P Campbell M Deidda M , et al. (2025) Using natural experiments to evaluate population health and health system interventions: New framework for producers and users of evidence. BMJ 388: e080505. 10.1136/bmj-2024-080505 PMC11950994 40154983

[bibr5-13563890261444326] Gates EF Walton M Vidueira P , et al. (2021) Introducing systems- and complexity-informed evaluation. New Directions for Evaluation 2021(170): 13–25.

[bibr6-13563890261444326] Hawe P Potvin L (2009) What is population health intervention research? Canadian Journal of Public Health 100(1): I8–114. 10.1007/BF03405503 PMC6973897 19263977

[bibr7-13563890261444326] Hawe P Shiell A Riley T (2004) Complex interventions: How ‘out of control’ can a randomised controlled trial be? BMJ 328(7455): 1561–1563. 10.1136/bmj.328.7455.1561 PMC437159 15217878

[bibr8-13563890261444326] Hawe P Shiell A Riley T (2009) Theorising interventions as events in systems. American Journal of Community Psychology 43(3–4): 267–276. 10.1007/s10464-009-9229-9 19390961

[bibr9-13563890261444326] Hovmand PS (2014) Introduction to community-based system dynamics. In: Community Based System Dynamics. Springer, pp.1–16. Available at: http://link.springer.com/10.1007/978-1-4614-8763-0_1 (accessed 28 November 2022).

[bibr10-13563890261444326] McGill E Er V Penney T , et al. (2021) Evaluation of public health interventions from a complex systems perspective: A research methods review. Social Science & Medicine 272: 113697. 10.1016/j.socscimed.2021.113697 33508655

[bibr11-13563890261444326] McGill E Marks D Petticrew M , et al. (2022) Addressing alcohol-related harms in the local night-time economy: a qualitative process evaluation from a complex systems perspective. BMJ Open 12(8): e050913. 10.1136/bmjopen-2021-050913 PMC9422880 36008081

[bibr12-13563890261444326] Meadows DH Wright D (2009) Thinking in Systems: A Primer. Earthscan.

[bibr13-13563890261444326] Moore GF Evans RE Hawkins J , et al. (2019) From complex social interventions to interventions in complex social systems: Future directions and unresolved questions for intervention development and evaluation. Evaluation 25(1): 23–45. 10.1177/1356389018803219 PMC6330692 30705608

[bibr14-13563890261444326] Ogilvie D Adams J Bauman A , et al. (2019) Using natural experimental studies to guide public health action: Turning the evidence-based medicine paradigm on its head. Journal of Epidemiology and Community Health. Epub ahead of print 19 November. 10.1136/jech-2019-213085 PMC6993029 31744848

[bibr15-13563890261444326] Rutter H Savona N Glonti K , et al. (2017) The need for a complex systems model of evidence for public health. The Lancet. Epub ahead of print 13 June. 10.1016/S0140-6736(17)31267-9 28622953

[bibr16-13563890261444326] Skivington K Matthews L Simpson SA , et al. (2021) A new framework for developing and evaluating complex interventions: Update of medical research council guidance. BMJ 374: n2061. 10.1136/bmj.n2061 PMC8482308 34593508

[bibr17-13563890261444326] Sniehotta FF Araújo-Soares V Brown J , et al. (2017) Complex systems and individual-level approaches to population health: A false dichotomy? The Lancet Public Health 2(9): e396–e397. 10.1016/S2468-2667(17)30167-6 29253408

[bibr18-13563890261444326] White M (2017) Protocol: Evaluation of the Health Impacts of the UK Treasury Soft Drinks Industry Levy (SDIL). Available at: https://www.journalslibrary.nihr.ac.uk/programmes/phr/1613001/#/ (accessed 18 June 2019).

